# Enhanced Fluidity of ZL205A Alloy with the Combined Addition of Al–Ti–C and La

**DOI:** 10.3390/ma14206169

**Published:** 2021-10-18

**Authors:** Guowei Zhang, Zhaojie Wang, Jingwei Niu, Hong Xu, Xiaoyan Ren

**Affiliations:** 1 School of Materials Science and Engineering, North University of China, Taiyuan 030051, China; wzj1997vip@163.com (Z.W.); niukingway@163.com (J.N.); xuhong@nuc.edu.cn (H.X.); 2 Department of Mechanical Engineering, Taiyuan Institute of Technology, Taiyuan 030008, China; renxiaoyan03@126.com

**Keywords:** ZL205A, fluidity, Al–Ti–C, La, grain refinement

## Abstract

The effects of Al–Ti–C and La on the fluidity of a ZL205A alloy after separate and combined addition were studied by conducting a fluidity test. The fluidity of the ZL205A alloy first increased and then decreased with the increasing addition of Al–Ti–C and La; it peaked at 0.3% and 0.1% for Al–Ti–C and La, respectively. The combined addition of Al–Ti–C and La led to better fluidity, which increased by 74% compared with the base alloy. The affecting mechanism was clarified through microstructure characterization and a DSC test. The heterogeneous nucleation aided by Al–Ti–C and La, the number of particles in the melt, and the evolution of the solidification range all played a role. Based on the evolution of the fluidity and grain size, the optimal levels of Al–Ti–C and La leading to both high fluidity and small grain size were identified.

## 1. Introduction

Aluminum alloys are widely used in the aircraft and automobile industry due to their low density and high strength [1,2]. The development of vehicle components requires aluminum alloys with higher strength and heat resistance. Al–Cu is a series of high–strength alloys with an Al–Cu phase precipitated during aging treatment [3]. ZL205A is an Al–Cu–based alloy and exhibits good mechanical properties; however, its wide freezing range leads to low fluidity, which may result in incomplete mold filling or casting defects during the production of its thin–walled components and the deterioration of its mechanical properties [4,5]. The fluidity of the alloy can be enhanced by optimizing the casting conditions, such as superheat, mold temperature, and pressure [6,7]; or by tailoring the solidification characteristics and thermophysical properties of the alloy [8,9,10,11]. However, the former approach may bring about other problems, so the latter is preferable.

Many studies reported the correlation between microstructure refinement/modification and enhanced fluidity [9,12]. Niu et al. [9] reported that Ce improves the fluidity of A356 alloy through the refinement of α–Al and the modification of eutectic Si. The mixed rare–earth addition of La and Yb refined the microstructure and modified the morphology of Al8Si6Mg3Fe, which increased its fluidity [13]. However, the addition of Cu to Al–Mg–Si alloy decreased its fluidity due to the refinement of the second phases, which increased surface area, leading to high flow resistance [14]. Prukkanon [12] reported the effect of the addition of Sc on the fluidity of A356 alloy and found a refined microstructure and increased fluidity after the addition of Sc; however, the alloy with the finest grain does not correspond to the highest fluidity. Since the alloying elements affect the fluidity of aluminum alloys by modifying the solidification range, the dendrite coherency temperature, and phase composition aside from microstructure refinement/modification [15], it is difficult to analyze the effect of grain refinement on the fluidity of aluminum alloys.

Al–Ti–C is a potent grain refiner for aluminum alloys, so Al–Ti–C is added to aluminum alloy to study the effect of grain refinement on the fluidity of aluminum alloy. Moreover, Ding et al. [16] found that the refining efficiency of the Al–Ti–C–La composite materalloy is more effective than without La, and Li et al. [6] found that La increases the fluidity of aluminum alloy. However, the combined effect of Al–Ti–C and La on the fluidity of aluminum alloy is not known. This information could aid in the development and application of grain refiners containing Al–Ti–C and La to achieve both high grain refinement potential and a positive effect on fluidity.

In this study, Al–Ti–C and La were added to the ZL205A alloy separately and synergistically to study their effect on the fluidity of the alloy. A special melt pouring system was designed for the fluidity measurement to ensure precise control over the velocity head and elevation head, which allowed a more accurate evaluation of the effect of alloy composition on the fluidity. The experimental results will benefit the optimization of fluidity of ZL205A alloy and provide insight into the composition design of other Al–Cu alloys.

## 2. Materials and Methods

**Figure 1:**
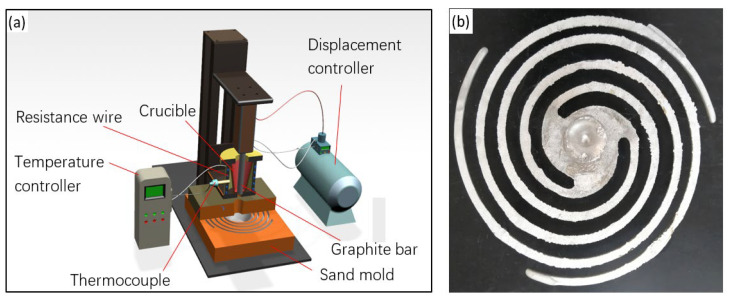
Schematic illustration of the fluidity test apparatus (**a**) and a typical spiral fluidity sample (**b**).

Pure Al, pure Cd, Al–50%Cu, Al–4%V, Al–10%Mn, Al–4%Zr, Al–10%La, Al–4%Ti–1%B and Al–5%Ti–0.5%C master alloys were used to prepare the experimental alloy (all compositions in this study were in wt.%). ZL205A has a composition of 4.95% Cu, 0.12% V, 0.49% Mn, 0.13% Zr, 0.25% Ti, 0.14% Cd, 0.01% Fe, 0.02% Si, and is balanced with Al. The content of Al–Ti–C varies between 0.1% and 0.7% and the La content ranges from 0.01% to 0.2%. Pure Al was melted in a resistance furnace under 730 °C. The alloying elements, Al–Ti–C, and Al–La master alloys were added, followed by mechanical stirring and degassing with C2Cl6.; the adopted melting process was typical of that used for Al–Cu alloys [4,17,18]. The melt was then poured into the crucible above the fluidity test mold, as shown in Figure 1a. After the temperature stabilized at 710 °C, the upward movement of the graphite bar controlled by the displacement controller led to the pouring of the melt into the sand mold. Figure 1b shows the typical fluidity test specimen; the length of the spiral fluidity sample was measured after the casting was cooled down to room temperature. The fluidity reported for each alloy is the average of at least two samples.

The metallographic specimens were cut from the spiral samples, ground, polished, and anodized with HFB4 or etched with 0.5% HF solution. An optical microscope (ZEISS, Oberkochen, German) and a scanning electron microscope (HITACHI, Tokyo, Japan) were used to characterize the microstructure of the alloys. The DSC measurement was conducted on the setaramLabsys (SETARAM, Lyon, France) with a temperature range of 20–800 °C, a heating rate of 10 °C/min, and a cooing rate of 20 °C/min under the protection of flowing Ar gas.

## 3. Results and Discussion

**Figure 2:**
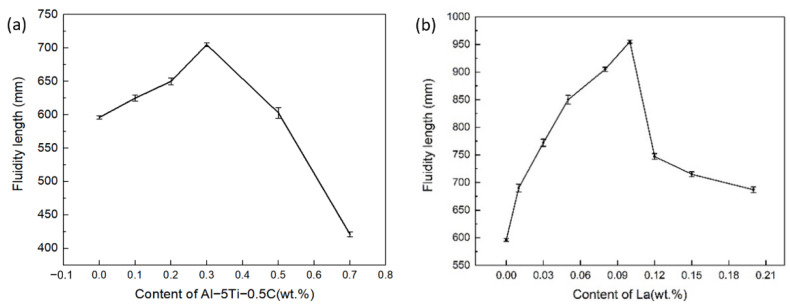
The fluidity of the ZL205A alloy with different levels of (**a**) Al–Ti–C and (**b**) La.

The evolution of the fluidity of the ZL205A alloy with the increasing content of Al–Ti–C is shown in Figure 2a. A non–monotonic relationship between the content of Al–Ti–C and fluidity can be observed. With the increasing content of the Al–Ti–C, the fluidity first increased and then decreased; it peaked at 0.3%, with an 18% increase in fluidity compared with the untreated ZL205A alloy. The effect of the addition of La on the fluidity is shown in Figure 2b: the fluidity first increased and then decreased with the increasing content of La. The optimal content was 0.1% within the experimental range in this study, which corresponded to a sharp increase in the spiral sample’s length from 596 mm of the untreated alloy to 955 mm, an increase of 60%.

**Figure 3:**
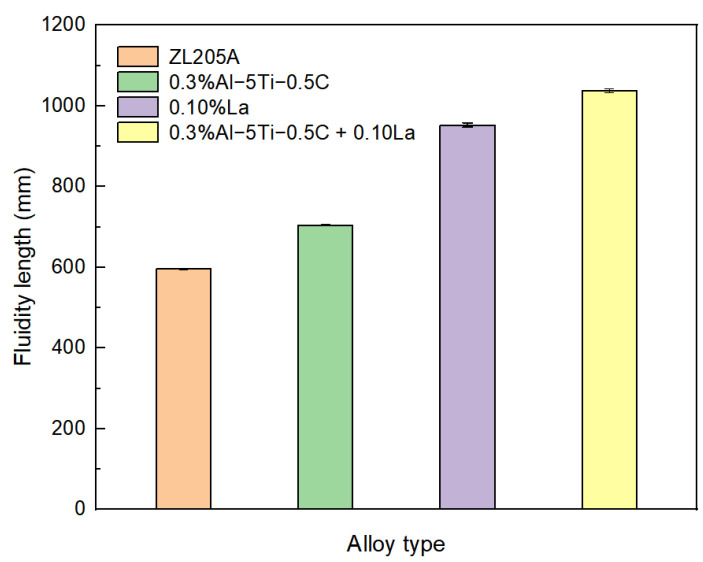
Effect of combined addition of Al–Ti–C and La on the fluidity of ZL205A alloy.

Considering the beneficial effects of both Al–Ti–C and La on the fluidity of the ZL205A alloy, the effect of the combined addition of Al–Ti–C and La was investigated. The content of Al–Ti–C and La was chosen to be 0.3% and 0.1%, respectively, in accordance with their optimal contents when added separately. The results are shown in Figure 3. The fluidity of the untreated ZL205A and the alloy with the addition of either Al–Ti–C or La are also plotted for comparison. The combined addition of Al–Ti–C and La demonstrated the highest fluidity, with an increase in the spiral sample’s length by 74% compared with the untreated alloy.

**Figure 4:**
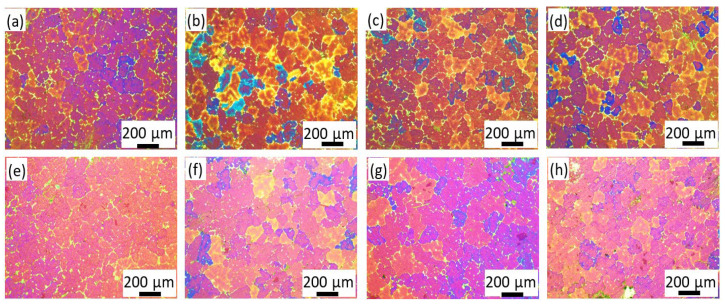
Microstructure of ZL205A alloy with different levels of Al–Ti–C or La: (**a**) ZL205A, (**b**) 0.1% Al–Ti–C, (**c**) 0.3% Al–Ti–C, (**d**) 0.7% Al–Ti–C, (**e**) 0.05% La, (**f**) 0.1% La, (**g**) 0.15% La, (**h**) 0.3% Al–Ti–C + 0.1% La.

**Figure 5:**
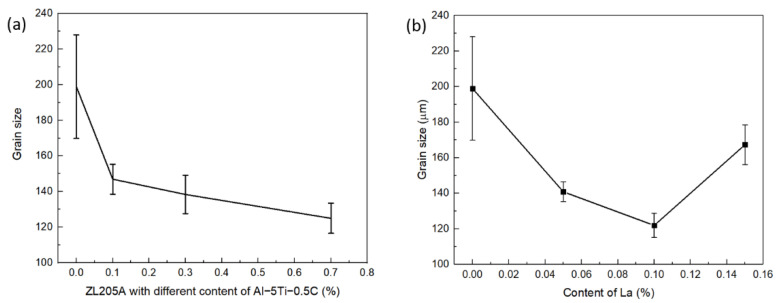
Evolution of grain size with the addition of (**a**) Al–Ti–C and (**b**) La.

To clarify the reason for the evolution of the fluidity with the addition of Al–Ti–C and La, and the synergistic effect of Al–Ti–C and La in enhancing the fluidity of the ZL205A alloy, the microstructure of the alloys was studied. ZL205A presented relatively coarse and inhomogeneous grains around 200 μm, as shown in Figure 4a, which resulted from the low cooling rate of the alloy in the sand mold. The addition of Al–Ti–C refined the microstructure, as shown in Figure 4b–d and Figure 5a. A sharp decrease in grain size was observed after the addition of 0.1% Al–Ti–C, and the grain size decreased gradually with the increasing content of Al–5Ti–0.5C. This was consistent with the phenomenon that nucleation is independent of the number of potent particles when its fraction exceeds a certain value [19]. The grain size first decreased and then increased with the increasing La content, and 0.1% La demonstrated the highest efficiency for grain refinement, as shown in Figure 4e–g and Figure 5b. The combined addition of 0.3% Al–5Ti–0.5C and 0.1% La demonstrated the most potent effect on grain refinement; the grain size decreased to 110 μm, as shown in Figure 4h.

The microstructure of the ZL205A fluidity test sample demonstrated equiaxed dendrite near the sand mold wall, as opposed to columnar dendrite; therefore, the solidification progressed not through the advancement of the planar interface, but in a bulk manner [20]. The low density of the nucleation site in the base alloy made a few nuclei grow rapidly and form grains with large sizes and developed dendrite due to the lack of high–solute nuclei around them. These large grains with highly developed dendrite structures contacted each other and hindered the flow of the melt, resulting in low fluidity. After the addition of Al–Ti–C or La, the Al3Ti and TiC in the Al–5Ti–0.5C acted as heterogeneous nucleation sites for α–Al; the addition of La also aided the nucleation of the grains by acting as a surfactant and decreased the contact angle between the nucleus and the substrate [10]. The increased number of nuclei led to overlapping solute fields and a reduced growth rate among the grains, which delayed the choke of the flow channel. This was consistent with what was found in the solidification of the ZL205A alloy under the traveling magnetic field, where the second dendritic arm and dendritic overlap were broken and the feeding channel widened [4], except that the formation of the large microstructure and dendritic overlap were restricted in the first place with the addition of Al–Ti–C and La. Based on the above analysis, smaller grains should have corresponded to high fluidity; however, this was not the case, as shown in Figure 2. To clarify the phenomenon, the evolution of the second phases in the alloy after the addition of Al–Ti–C and La were analyzed.

**Figure 6:**
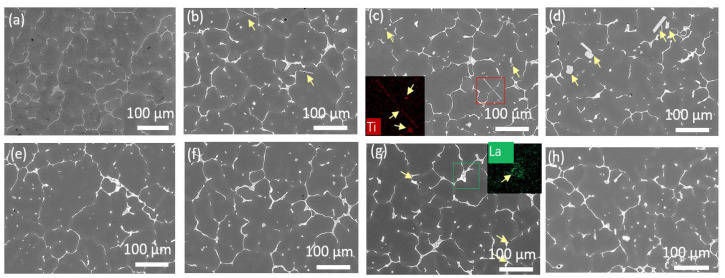
Microstructure of ZL205A alloy with different levels of Al–Ti–C or La: (**a**) ZL205A, (**b**) 0.1% Al–Ti–C, (**c**) 0.3% Al–Ti–C, (**d**) 0.7% Al–Ti–C, (**e**) 0.05% La, (**f**) 0.1% La, (**g**) 0.15% La, (**h**) 0.3% Al–Ti–C+0.1% La. The inset in (**c**,**g**) is the EDS mapping of the highlighted region.

Needle–shaped or blocky Al3Ti and dotted TiC were observed after the addition of Al–Ti–C, and their fraction increased with increasing content of Al–Ti–C, as represented by the arrows in Figure 6b–d. No new phase was observed in the La–containing alloy when the concentration of La was below 0.1%, as shown in Figure 6e,f. A new phase with bright contrast was observed when the La concentration was 0.15% as highlighted by the arrows in Figure 6g. The EDS results indicated that the phase contained La elements, which are thought to be Al11La3, according to the phase diagram. The Al11La3 phase formed through the Al–La eutectic reaction at a temperature higher than that of the Al–Cu eutectic transformation [21]. The Al11La3 phase, TiC, and Al3Ti hindered the melt flow due to the stagnant boundary layer around these particles [22]. The detrimental effect of these particles on the fluidity was compensated by the grain refinement effect of Al3Ti, TiC and the La elements when the concentration of the particles was low and the grain refinement effect was clear, i.e., when the Al–Ti–C was below 0.3% and the concentration of La was lower than 0.1%. With a further increase of Al–Ti–C or La, the number of particles increased, as shown in Figure 6d,g, but without an apparent further decrease in grain size; therefore, the hindrance to the fluid flow increased and resulted in decreased fluidity. This explains the peaks in the fluidity–content curves of the ZL205A alloy, shown in Figure 2a,b.

**Figure 7:**
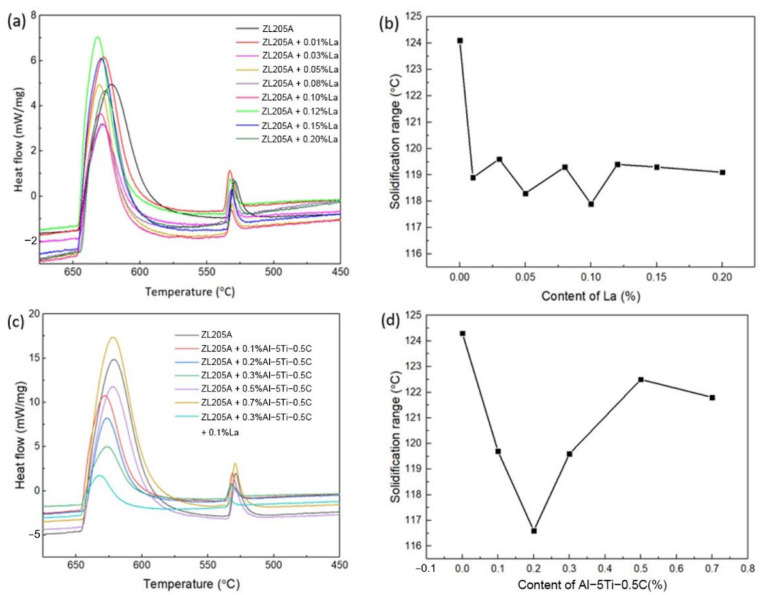
DSC cooling curves and solidification range of ZL205A alloy with different levels of (**a**,**b**) La and (**c**,**d**) Al–Ti–C.

Since the solidification range had a significant effect on the fluidity of the aluminum alloys, the effect of Al–Ti–C and La on the solidification range was studied. Figure 7 shows the DSC curves of the ZL205A alloy with the addition of Al–Ti–C and La. Two exothermic peaks were observed during cooling. The first resulted from the formation of α–Al and the second represents eutectic transformation; no additional peak was detected between these two peaks. The temperature difference between the two peaks, i.e., the solidification range, is plotted in Figure 7. It decreased by 5 °C after the addition of 0.1% La and fluctuated within ~2 °C with the increasing La content. This was consistent with the results obtained by Yang et al. [21], in which La decreased the solidification range of the Al–Cu alloy by increasing the eutectic temperature. The solidification range first decreased and then increased with the increasing content of Al–Ti–C, and the addition of Al–Ti–C within the range of 0.1% to 0.3% apparently decreased the solidification range; however, with further increases in the Al–Ti–C content, the solidification range increased. It is postulated that the decrease of the solidification range with a low content of Al–Ti–C resulted from the nucleation of the eutectic phase in the pre–existing Al3Ti and TiC phase, which decreased the eutectic temperature. However, with the increasing content of Al–Ti–C, the concentration of the Al3Ti and the TiC became oversaturated and their effect became less important; an increased number of Ti elements in the liquid just before the eutectic transformation decreased the eutectic transformation temperature, so the solidification range increased. This was consistent with the decrease in the Al–Cu’s eutectic temperature after the addition of 0.01% TiC [23]. The combined addition of Al–Ti–C and La resulted in a solidification range of 117 °C, a reduction of 7 °C compared with the ZL205A alloy. Since the size of the mushy zone was dependent on the solidification range and the temperature gradient and the narrow solidification range led to a smaller mushy zone and better fluidity under a similar temperature distribution [18], the reduced range of the crystallization temperature of the ZL205A alloy with the addition of La and Al–Ti–C resulted in higher fluidity.

The Al–Ti–C and La imposed their effect on fluidity by changing the solidification characteristics. Heterogeneous nucleation aided by Al–Ti–C and La elements delayed the formation of grains that were large enough to interconnect and choke the flow channel. The suppression of large grains with developed dendrite with the addition of Al–Ti–C and La came at a cost: excessive TiC, Al3Ti and La particles led to a higher solid–liquid surface area, which increased the resistance of melt flow. Therefore, the beneficial effect of fluidity resulting from grain refinement was first counteracted and then overcome by the adverse effect of the excessive solid phase, which was due to the increased levels of Al–Ti–C and La. This effect combined with the evolution of the solidification range with the addition of the Al–Ti–C and La and made the ZL205A with 0.3% Al–Ti–C and 0.1% La exhibit the highest fluidity.

Although the grain size decreased with the increasing content of Al–Ti–C within the experimental range in this study, the concentration–grain size curve flattened and the fluidity dropped sharply when its content exceeded 0.3%. For the La element, 0.1% corresponded to both the highest fluidity and the smallest grain size within the experimental range. Therefore, the optimal levels of Al–Ti–C and La that lead to high fluidity and small grain size are 0.3% and 0.1% respectively.

## 4. Conclusions

The effect of Al–Ti–C and La on the fluidity of a ZL205A alloy was studied by conducting a fluidity test. DSC and microstructure characterization were carried out to clarify the mechanism through which they influenced the fluidity under separate and combined addition. The following conclusions are drawn.

The fluidity of the ZL205A alloy first increased and then decreased with the increasing addition of Al–Ti–C and La; it peaked at 0.3% and 0.1% for the Al–Ti–C and the La, respectively. The combined addition of Al–Ti–C and La led to better fluidity.

The optimal levels of Al–Ti–C and La that led to both high fluidity and small grain size were 0.3% and 0.1%, respectively, which corresponded to an increase of 74% in fluidity compared with the untreated ZL205A alloy.

The suppression of the formation of large grains with developed dendrite structures and the decrease in the solidification range by Al–Ti–C and La contributed to the enhanced fluidity.

## Data Availability

The data are not publicly available due to it is part of another paper.
